# Supplementary material for: Development of a computer-aided design software for the quantitative evaluation of aesthetic damage
Source: PLoS One. 2019 Dec 18;14(12):e0226322. doi: 10.1371/journal.pone.0226322 (PMC6919621; doi:10.1371/journal.pone.0226322)
Supplement: S1 Code site — 1. (ZIP) [file pone.0226322.s001.zip › [EESC_jr][Projeto]Relatorio_07_21/reports/templates_zip/gentelella-master/vendors/morris.js/examples/negative.html]

# Negative values

```
var neg_data = [
  {"period": "2011-08-12", "a": 100},
  {"period": "2011-03-03", "a": 75},
  {"period": "2010-08-08", "a": 50},
  {"period": "2010-05-10", "a": 25},
  {"period": "2010-03-14", "a": 0},
  {"period": "2010-01-10", "a": -25},
  {"period": "2009-12-10", "a": -50},
  {"period": "2009-10-07", "a": -75},
  {"period": "2009-09-25", "a": -100}
];
Morris.Line({
  element: 'graph',
  data: neg_data,
  xkey: 'period',
  ykeys: ['a'],
  labels: ['Series A'],
  units: '%'
});
```
